# Giant Intra-Abdominal Desmoid Tumor in a Young Male without History of Surgery, Trauma, or Familial Adenomatous Polyposis

**DOI:** 10.1155/2018/9825670

**Published:** 2018-09-04

**Authors:** Noritoshi Mizuta, Kozo Tsunemi

**Affiliations:** Department of Surgery, Akashi Medical Center, Hyogo, Japan

## Abstract

Desmoid tumors are rare, monoclonal myofibroblastic neoplasms that occur in the extremities, the trunk, and the abdominal cavity. We present a case that is significant for its rarity and for consideration of its treatment plan. A 17-year-old male reported swelling of his abdomen and abdominal pain. He was referred to our hospital with no history of surgery, trauma, or familial adenomatous polyposis. A large tumor in the abdominal cavity was detected by computed tomography, and surgical resection was performed. The tumor was thought to have developed from the anterior lobe of the transverse colon mesentery. It weighed 5.9 kg. Tumor cells with collagen fibers were observed in histopathological examination, but heteromorphism and the nuclear fission image were not apparent. Immunostaining revealed beta-catenin expression in the tumor cell nucleus. Diagnosis was an intra-abdominal desmoid tumor. Currently, there are no signs of recurrence. In this case, preoperative diagnosis was difficult, but surgery was the optimal treatment according to the symptoms. Desmoid tumors have invasive development and common local recurrence, so sufficient range of resection including normal tissue and strict follow-up are necessary.

## 1. Introduction

Desmoid tumors (DTs) are benign, deep-seated monoclonal myofibroblastic neoplasms that slowly grow, infiltrate, and arise from musculoaponeurotic stromal elements [1]. They are rare; the incidence in the general population is 2–4 cases per million people per year [1]. DTs are typically sporadic and can occur anywhere in the body [1, 2]. They are reportedly associated with surgery, trauma, pregnancy, and familial adenomatous polyposis (FAP) [1, 2].

A giant intra-abdominal desmoid tumor (DT) developed in a young male without history of surgery, trauma, or FAP. The tumor contacted both his stomach and pancreatic tail. We performed surgical resection of the tumor with partial resection of the stomach and pancreatic tail. DTs have invasive development, and many recur locally, so it is thought that complete resection with a negative margin is important [1, 2].

## 2. Case Presentation

**Figure 1:**
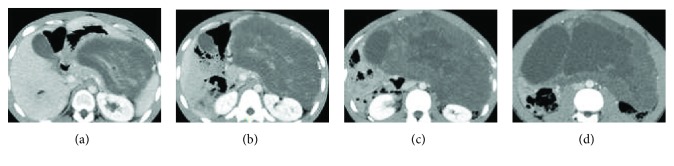
(a–d) CT scan with intravenous contrast (sagittal view): the tumor seemed to be divided into two parts. The right part was like a single cystic lesion; the left part contained a solid component.

**Figure 2:**
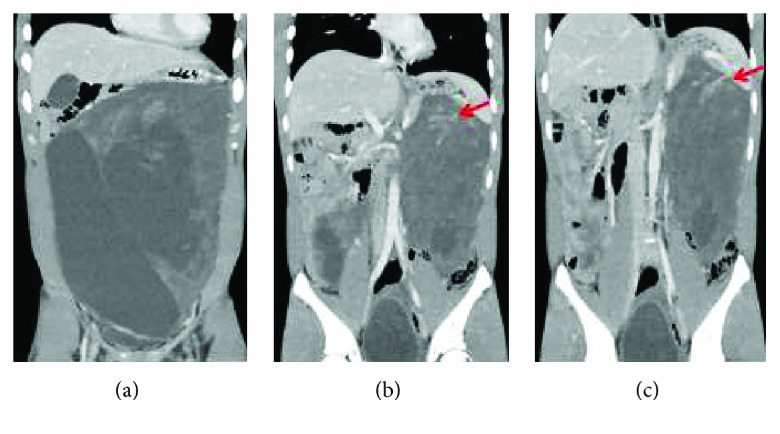
(a–c) CT scan with intravenous contrast (coronal view): the vessel seen from the left gastroepiploic artery to the tumor (red arrow).

**Figure 3:**
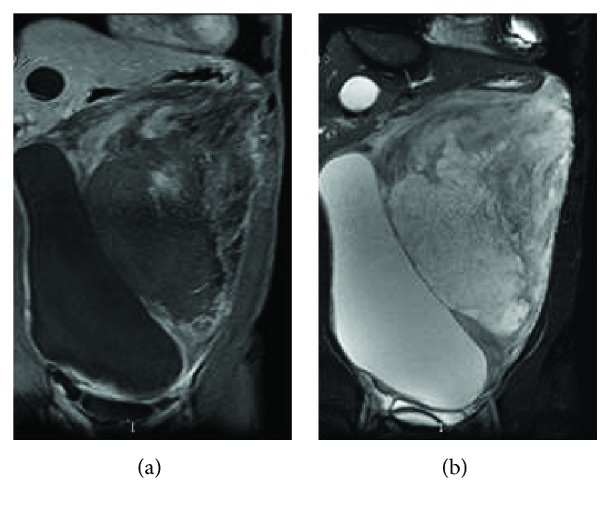
(a) MRI (coronal view, T1W1): tumor had generally low signal density, but some parts had high signal density. (b) MRI (coronal view, T2W1): tumor had high signal intensity.

A 17-year-old male noticed swelling of his abdomen from six months previously. He reported pain at the left side of the umbilicus. Body weight increased by 5 kg in one year. Computed tomography (CT) was performed at another hospital. A larger abdominal tumor was detected, so he was referred to our hospital for examination. Vital signs and laboratory data were normal, but the abdomen was bulging slightly. CT showed a giant tumor occupying the majority of the abdominal cavity (Figures 1(a)–1(d) and 2(a)–2(c)). The tumor seemed to be divided into two parts. One part was a single cystic lesion, which had no contrast effect from the right abdomen to the pelvic cavity. The other part, from left upper abdomen to the lower abdomen, appeared to have a solid component where the contrast effect was mild. The vessel was seen from the left gastroepiploic artery to the tumor. Magnetic resonance imaging (MRI) showed the tumor had almost entirely low signal density, but T1-weighted image (T1WI), some parts had high signal density (Figure 3(a)). In T2-weighted image (T2WI), on the other hand, the tumor showed high signal intensity (Figure 3(b)).

On the gadolinium-enhanced image, the contrast effect was poor, and the high signal area was only slight.

Preoperative diagnosis was a giant abdominal cystic tumor. Differential diagnosis was gastrointestinal stromal tumor (GIST), DT, or lymphangioma.

Surgery was performed for definitive diagnosis and improvement of the symptoms.

### 2.1. Operative Findings

**Figure 4:**
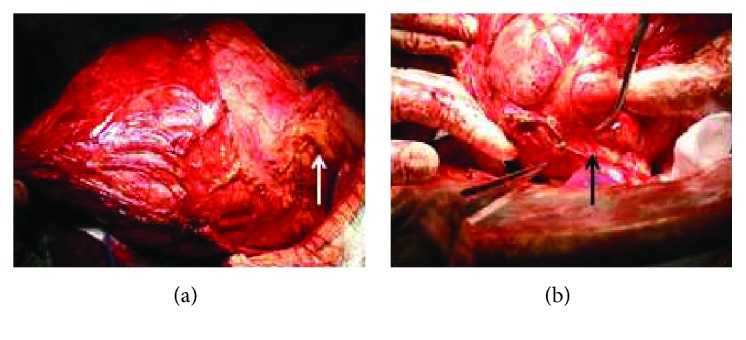
(a) Operative findings: the tumor was attached to the stomach and omentum (white arrow). (b) Operative findings: tumor also adhered to the pancreatic tail (black arrow).

A lower midline incision was performed to observe the large tumor contained within a membrane (Figure 4(a)). The tumor was found to be adhered to both the mesocolon and omentum, and blood vessels were observed to be coming from both; angered vessels bled easily. Fluid in the right part of the tumor was aspirated; about 1.7 L greenish-brown fluid was collected. We separated the tumor from the mesocolon. The omentum artery of the stomach was preserved, and the omentum was also separated from the tumor. In this time, we judged that the tumor originated from the anterior lobe of the transverse mesocolon. The tumor was in contact with the great curvature of the stomach. The adhesion could not be separated, so we partially resected the stomach. The branches from the left gastroepiploic artery had become thick, indicating it was probably the main feeder of the tumor. Furthermore, the tumor was also strongly in contact with the pancreatic tail (Figure 4(b)). The border line was unclear. If the pancreatic tail was preserved, the capsule of the tumor remained, and the possibility of recurrence existed. So pancreatic tail resection was performed, and the tumor was excised. The tumor itself was 4.2 kg, and aspirated fluid was 1.7 L, so the total tumor weight was 5.9 kg. Operation time was 2 hr 32 min, and bleeding volume was 220 mL.

### 2.2. Specimen

**Figure 5:**
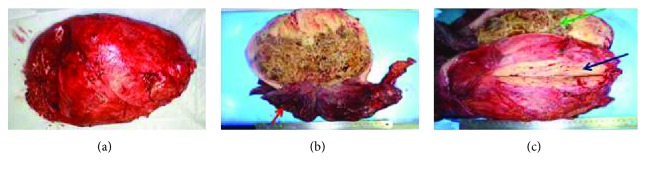
(a) Specimen: size was 30 × 25 × 10.5 cm. (b) Specimen: right side of the tumor was a cystic component with wall thickening (red arrow). (c) Specimen: when tumor was incised, there was a mixture of solid components (blue arrow) and parts of honeycomb-like texture (green arrow).

The tumor is shown in Figures 5(a)–5(c). It measured 30 × 25 × 10.5 cm. On the right side of the tumor, a cystic component with wall thickening was found. When the tumor was incised, there was a mixture of solid components and parts that showed honeycomb-like texture.

**Figure 6:**
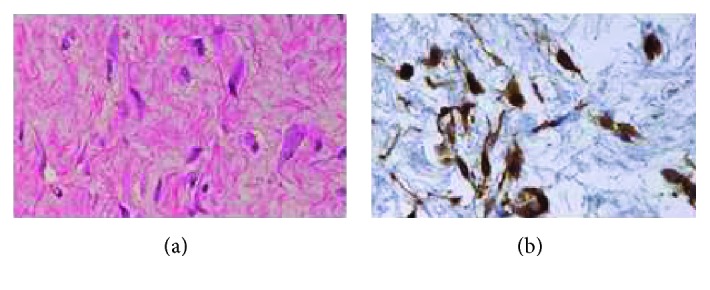
(a) Specimen (HE staining): spindle-shaped or star-shaped tumor cells proliferate diffusely with abundant collagen fiber. (b) Specimen (beta-catenin staining): beta-catenin was positive in the tumor cell nucleus.

Pathological findings included spindle-shaped or star-shaped tumor cells proliferating diffusely with abundant collagen fiber. Heteromorphism was not noticeable, and the nuclear fission image was not apparent. Beta-catenin was positive in the tumor cell nucleus on immunohistochemistry (Figures 6(a) and 6(b)).

Final diagnosis was an intra-abdominal desmoid tumor.

### 2.3. Postoperative Course

Postoperative pancreatic fistula occurred but was improved with nonoperative therapy. The patient was discharged on the 16th postoperative day. After discharge, colonoscopy (CS) was unremarkable. The patient is now being followed-up as an outpatient.

## 3. Discussion

DTs are benign, deep-seated monoclonal myofibroblastic neoplasms that slowly grow, infiltrate, and arise from musculoaponeurotic stromal elements [1]. DTs are rare; they account for only 0.03% of all neoplasms and less than 3% of all soft tissue tumors [1]. They can arise from anywhere on the body but are generally classified into three main anatomic locations: extra-abdominal (trunk and extremities), along the abdominal wall, and least commonly, intra-abdominally [3]. The most likely location for intra-abdominal DTs is the mesentery, especially the small bowel [3]. DTs are slightly more common in women than men, with some DTs related to pregnancy and trauma and others associated with hereditary cancer syndromes [1]. For example, 5~15% of DTs are associated with FAP [1, 4]. Patients with FAP have a more than 800-fold risk of developing DTs compared to the general population [4].

For diagnosis of DTs, especially intraabdominal DTs, representative symptoms are intestinal obstruction, bowel ischemia, and abdominal distention [1]. Many patients with intra-abdominal DTs who had no family history of colon cancer and no personal history of abdominal trauma receive diagnosis without symptoms [2]. If the patients have history of FAP and abdominal trauma, the DTs can easily be given differential diagnosis. Our case is a rarity for two reasons; the DT was intra-abdominal, and the patient had no specific medical history.

Concerning imaging study, both CT and MRI play important roles. On CT, DTs usually appear as a well-circumscribed homogenous lesion isodense or hyperdense relative to muscle, although GIST may have a similar appearance [5]. Differential diagnosis of such a mesenteric cystic mass lesion are carcinoid, leiomyoma, leiomyosarcoma, and lymphoma [5]. Contrast enhancement is variable, most DTs demonstrating mild-to-moderate enhancement [6].

The tumor in our patient had two parts; one part was a single cystic lesion without contrast enhancement, and the other part seemed to have a solid component inside, the contrast effect of which was mild. About the MRI, signal intensity is reflective of the proportion of collagen fibers, spindle cells, and extracellular matrix present [6]. Most intra-abdominal DTs have low or intermediate signal intensity on T1WI and have heterogeneous low to high signal intensity on T2WI [5–7].

The contrast enhancement pattern is variable, moderate-to-marked enhancement [6, 7]. Although providing useful information, neither CT nor MRI images can fully rule out or confirm DTs; for definitive diagnosis, a biopsy or surgical resection is necessary.

Regarding treatment, the optimal therapy for DTs is also difficult to ascertain. DTs are rare, and anatomical presentation is varied, so large randomized trial studies are difficult [1, 2, 8]. Close observation is an acceptable strategy for stable asymptomatic patients (watchful waiting) [1–3, 5, 8]. If the patient has symptoms, however, the optimal therapy is complete surgical resection with negative margin when medically and technically feasible [2, 3]. Our patient had symptoms, but definitive diagnosis was not possible, so surgical resection was the optimal treatment. Additional partial resection of the stomach and pancreatic tail was appropriate because the tumor adhered to both organs.

In spite of the complete resection of the tumor, the recurrence rate of the DTs ranges between 30 and 40% [1]. The absence of any impact of the positive microscopic margin on patient outcome was confirmed in a previous article [9]. Surgical resection should therefore be performed with function preservation to minimize major morbidity. In our case, if the progression of the tumor to the other organs was more severe, complete resection may have led to serious complications. To prevent this, if we encounter a similar case in the future, preoperative biopsy from the tumor may also be taken into account. There is also possibility of dissemination, so accumulation of the similar cases is necessary for analysis. If the tumor recurs, radiation and systemic therapy, such as tamoxifen, doxorubicin, nonsteroidal anti-inflammatory drugs, and interferon, are suitable [1, 2, 8].

In summary, we encountered a rare case of giant intra-abdominal DT in a young male who had no history of trauma, surgery, or AFP. Although he had symptoms, definitive diagnosis was not possible, so optimal treatment was surgery. DTs have a high rate of recurrence, so complete resection and close follow-up are necessary.
